# In Situ Thermal Generation of Silver Nanoparticles in 3D Printed Polymeric Structures

**DOI:** 10.3390/ma9070589

**Published:** 2016-07-19

**Authors:** Erika Fantino, Annalisa Chiappone, Flaviana Calignano, Marco Fontana, Fabrizio Pirri, Ignazio Roppolo

**Affiliations:** 1 Department of Applied Science and Technology, Politecnico di Torino, Corso Duca degli Abruzzi, 24, Torino 10129, Italy; erika.fantino@polito.it (E.F.); marco.fontana@polito.it (M.F.); fabrizio.pirri@polito.it (F.P.); 2 Center for Sustainable Futures@PoliTo, Istituto Italiano di Tecnologia, Corso Trento, 21, Torino 10129, Italy; flaviana.calignano@iit.it (F.C.); ignazio.roppolo@iit.it (I.R.)

**Keywords:** 3D printing, polymer-based nanocomposites, silver nanoparticles

## Abstract

Polymer nanocomposites have always attracted the interest of researchers and industry because of their potential combination of properties from both the nanofillers and the hosting matrix. Gathering nanomaterials and 3D printing could offer clear advantages and numerous new opportunities in several application fields. Embedding nanofillers in a polymeric matrix could improve the final material properties but usually the printing process gets more difficult. Considering this drawback, in this paper we propose a method to obtain polymer nanocomposites by in situ generation of nanoparticles after the printing process. 3D structures were fabricated through a Digital Light Processing (DLP) system by disolving metal salts in the starting liquid formulation. The 3D fabrication is followed by a thermal treatment in order to induce in situ generation of metal nanoparticles (NPs) in the polymer matrix. Comprehensive studies were systematically performed on the thermo-mechanical characteristics, morphology and electrical properties of the 3D printed nanocomposites.

## 1. Introduction

Polymer-based nanocomposites (NCs) have been extensively studied in the last decades as a means to achieve improved properties via the control of the interactions between the polymeric host and the nanostructured filler [1,2]: They have become an important class of materials exploited in many different applications such as optics [3,4,5,6], microelectronics [7,8,9], bioactive materials [10,11] and others [12].

There are many nano- and micro-fabrication techniques available for the realization of such type of NCs, including electron beam lithography, photolithography, ink-jet printing, direct-write techniques, soft lithography and contact printing [13]. But these techniques do not offer simple approaches to fabricate three-dimensional (3D) structures.

Currently, great efforts are being produced in the attempt to develop new nanocomposite processing techniques that may allow the production of highly reliable and precise 3D microstructures: A very promising and potentially cost-effective approach to manufacture such nanocomposite microdevices is represented by 3D printing [14,15,16,17]. A marriage of nanomaterials and 3D printing could offer clear advantages and numerous new opportunities [18,19,20].

3D printing consists of the direct fabrication of a 3D object starting from a digital model. 3D printing is now routinely used in a variety of manufacturing sectors ranging from simple prototypes to direct part production, in both aerospace, automotive and bioengineering sectors [18,21,22,23].

In recent years, advances in materials science in conjunction with the existing 3D printing technologies are opening new areas of application: Researchers have investigated hybrid 3D printing processes and materials to create advanced products [15,16,24,25,26,27,28,29]. The possibility of coupling the 3D aspect with a low-cost fabrication would open up several possibilities in a broad range of fields, in particular in the electronic one [17,30,31].

Stereolithography (SLA) and Digital Light Processing (DLP) represent some of the most explored 3D techniques used for the fabrication of such microdevices [15,32]. The general procedure for building 3D structures with SLA involves the exposure of light (typically from a laser or light-emitting diode) to a photocurable resin (e.g., acrylated monomer or oligomer), which creates cross-linked regions where the light irradiates the matrix. The resolution of these techniques is influenced by many factors, depending both on the photocurable system (curing mechanism, kinetics, free radical diffusion, etc.) [33,34,35] and the optical system [36]. The throughput of the SLA process is slow due to the point-by-point scanning nature of the direct-write of the laser system while the DLP exploits a digital micro mirror-array device (DMD), to produce a dynamic digital mask: An entire part cross section can be cured at one time, resulting in a faster process than scanning a laser beam [36].

A significant amount of work on 3D printing of nanocomposites concerns the incorporation of different types of nanoparticles in a commercially available acrylate or epoxy resin. In most of the works the scope is to enhanced the properties of the matrix (electrical [37], magnetic [38], mechanical [39] and thermal properties [40]), instead in other papers the aim is to fabricate green bodies of ceramic components [41,42,43]. Nevertheless, in all the cases, the addition of nanofillers strongly affects the printing process: Solution viscosity, light penetration depth and nanoparticles dispersion and stability. The control over all these parameters can be really tedious and not easily achievable.

Recently a new method to obtain 3D polymer-based NCs was proposed [44]: The results established a novel approach for the preparation of 3D nanocomposites by coupling the photoreduction of metal precursors with the DLP technology, allowing the fabrication of conductive 3D hybrid structures consisting of metal nanoparticles and organic polymers shaped in complex multilayered architectures. The main advantage of this technique is the combination of the DLP technology with the reduction of the silver precursor.

**Figure 1:**
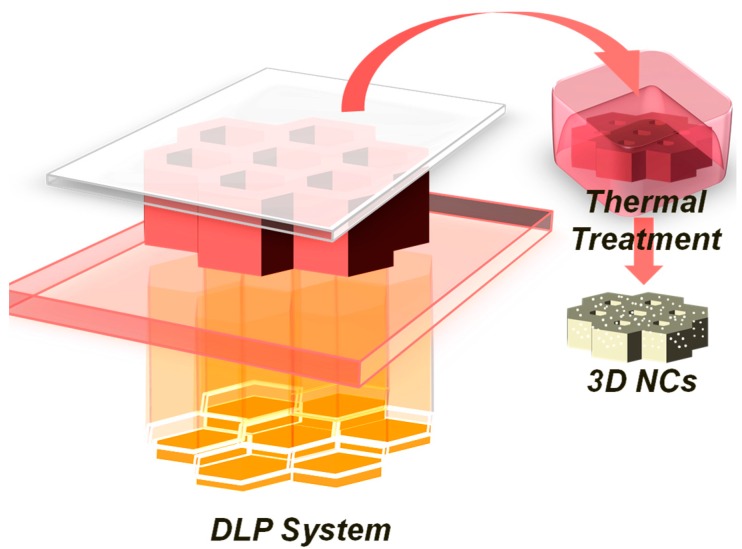
Sketch of Digital Light Processing (DLP) setup that projects dynamic digital masks on the photocurable formulation featuring the formation of the polyethylene glycol diacrylate structure. Subsequent Thermal treatment, with the formation of the silver nanoparticles by reduction of the metal precursors.

Based on previous studies [44], we select the best formulation and in this paper we decided to exploit a thermal reduction of the metal precursor evaluating the possibility of contemporary sintering of the generated silver nanoparticles. The 3D structures were fabricated by embedding metal salts in the starting formulation, PEGDA oligomer and photoinitiator, and exposing them to the DLP system. The 3D fabrication is then followed by a thermal treatment (TT), in order to induce the in situ generation of metal nanoparticles (NPs) in the polymer matrix, (Figure 1). Comprehensive studies were systematically performed on the thermo-mechanical characteristics, the morphology and the electrical properties of the 3D printed nanocomposites.

## 2. Experimental

### 2.1. Materials

PEGDA with a molecular weight of 700 g·mol^−1^ and AgNO_3_ were purchased from Sigma–Aldrich Srl (Milan, Italy) and used as received. Bis-(2,4,6-trimethylbenzoyl) phenylphosphineoxide (Irgacure 819, kindly provided by BASF, Kaisten, Switzerland) was selected for his fair absorbing characteristics in the deep blue to near UV, and was added to the formulation (1 phr). The dye selected, Reactive Orange (RO), was purchased from Sigma–Aldrich and used as received.

### 2.2. Preparation of the 3D Structures

A 3DLPrinter-HD 2.0 (Robot Factory, Mirano, Italy) was employed as printing equipment using a projector with a resolution of 50 μm (1920 × 480 × 1080 pixels). The build area is 100 × 56.25 × 150 mm^3^ and the layer thickness is adjustable from 10 to 100 μm. The exposure time was set at 1 s per layer for neat PEGDA and was increased up to 1.2 s per layer for the sample containing 15 phr of silver nitrate.

### 2.3. Thermal Treatmeant

The printed parts were submitted to thermal treatments in oven at 100, 150 and 200 °C; a Buchi Glass Oven (BÜCHI Labortechnik AG, Flawil, Switzerland) was employed for the thermal treatments perfomed in vacuum.

### 2.4. Characterization

DSC measurements were performed with a DSC1 STARe System apparatus of TA Instruments (TA Instruments Waters LLC, New Castle, DE, USA) equipped with a low temperature probe. The experiments were carried out between −80 and 60 °C with a heating rate of 10 °C·min^−1^. TGA was performed in air using a TGA/SDTA 851e instrument in the range between 25 and 700 °C, with a heating rate of 10 °C·min^−1^. The morphological characterization of the nanocomposite was carried out by FESEM (Zeiss Supra 40) (Carl Zeiss AG, Jena, Germany). The samples were prepared by fracturing the obtained 3D structures in liquid nitrogen: Both surface and cross-section of the cured materials were analyzed.

The UV–Visible spectra were recorded by means of a double beam Lambda 40 instrument (Perkin-Elmer Italia, Milano, Italy). The range between 280 and 800 nm was monitored with a scan rate of 480 nm/min. All the experiments were performed on 100 µm films coated on a glass slide.

Electrical conductivity of the 3D structure was measured by using a Keithley-238 High Current Source Measure Unit (Keithley Instruments, Cleveland, OH, USA) (voltage range ±50 V, step 1 V) realizing a two-point contact setup placing copper electrodes on the two opposite sides of flat specimens (area 0.5 cm^2^). The best performing sample was measured in more complex geometries. The data shown are obtained by multiple measurements on different samples (three for each formulation).

## 3. Results and Discussion

3D conductive polymeric structures were fabricated by incorporating silver nanoparticles precursor (Silver nitrate (AgNO_3_)) into photocurable polymer formulations based on polyethylene glycol diacrylate (PEGDA) and exposing them to DLP system. The formulations were prepared by dissolving a fixed concentration of AgNO_3_ (15 phr), and photoinitiator Irgacure 819 (1 phr) in the PEGDA oligomer. The DLP system works with an illumination system in the UVA-visible range so we properly selected a typical photoinitiator working in the near UV-violet-blue spectrum: Irgacure 819 that belongs to BAPO (Bis-Acyl-Phosphine Oxide) family. A dye (0.2 phr) was also added to the formulation since the bright color of the dye can prevent the leaking out of light from the desired illumination area and allows to control the thickness of each layer during the printing process.

**Figure 2:**
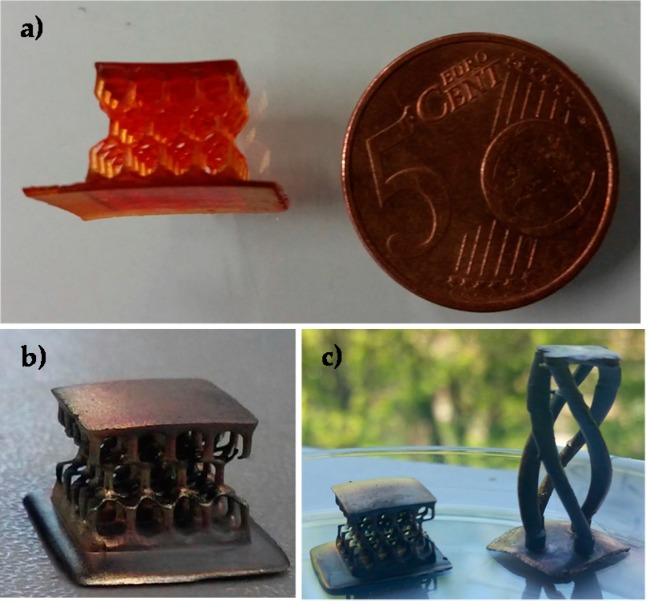
3D objects produced by DLP technique from the formulation containing polyethylene glycol diacrylate (PEGDA) and 15 phr of silver nitrate. (**a**) Honeycomb structure as printed; (**b**,**c**) samples after the thermal treatments; the metallic aspect induced by the presence of the silver nanoparticles is clearly visible.

Different computer-aided design (CAD) files were produced aiming to print different 3D objects, ranging from simple rectangular structure to more complex honeycomb and helicoidally structures (Figure 2).

After the realization of the 3D objects, different thermal treatments were performed in order to induce the reduction of the metal precursor, and the formation of the metal nanoparticles inside the printed structures.

According to the literature, sintering process of silver NCs are commonly performed at a temperature of about 200 °C [45]; the thermal treatments were performed at different temperatures up to 200 °C both in air and in vacuum aiming to obtain the reduction and to help the possible sintering of the NPs.

**Figure 3:**
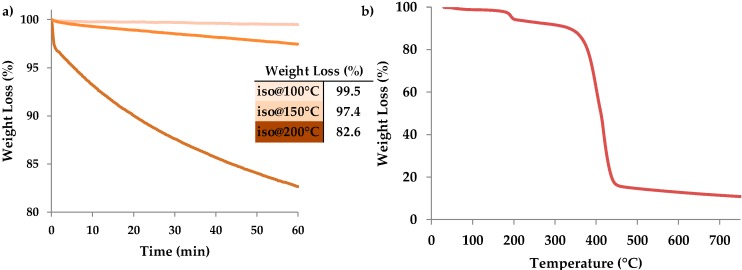
(**a**) Isothermal treatments performed in air at different temperature (100 °C, 150 °C and 200 °C); (**b**) thermogravimetric analysis (TGA) plot of the sample PEGDA_15 phr AgNO_3_ heated in nitrogen at a rate of 10 °C/min.

Firstly, in order to evaluate the best temperature window for the post process and to check its compatibility with 3D printed nanocomposites, thermogravimetric analysis (TGA) was performed on just printed samples containing silver precursors in order to follow the response of the 3D structure. Isothermal treatments performed in air confirmed that 200 °C thermal process is not compatible with our structures since the polymer matrix undergoes thermo-oxidative degradation, showing a loss of weight of 18% (Figure 3a). Thermal treatments performed at lower temperatures showed considerably lower loss of weight (0.2% for 100 °C treatment and 2.3% for 150 °C treatment), confirming that between 150 and 200 °C thermo-oxidative degradation occurs. TGA analyses were also conducted in inert atmosphere in order to simulate vacuum treatment since in vacuum or N_2_ atmosphere only thermal degradation could occur. The nanocomposite showed thermal stability up to 220 °C (Figure 3b) indicating that the post treatment could be performed up to 200 °C in vacuum. At higher temperature instead thermal polymer degradation occurs, leaving a final residue at higher temperature related to silver salts dispersed in the polymer matrix.

While 3D printed objects containing nitrate are heated, an irreversible color change is observed: The object evolves from a red color, (Figure 2a), to a dark brown until, for higher treatment temperatures, it reaches a silver mirror aspect (Figure 2b). This can be related to a nucleation-growth mechanism of the silver nanoparticles within the matrix [46].

**Figure 4:**
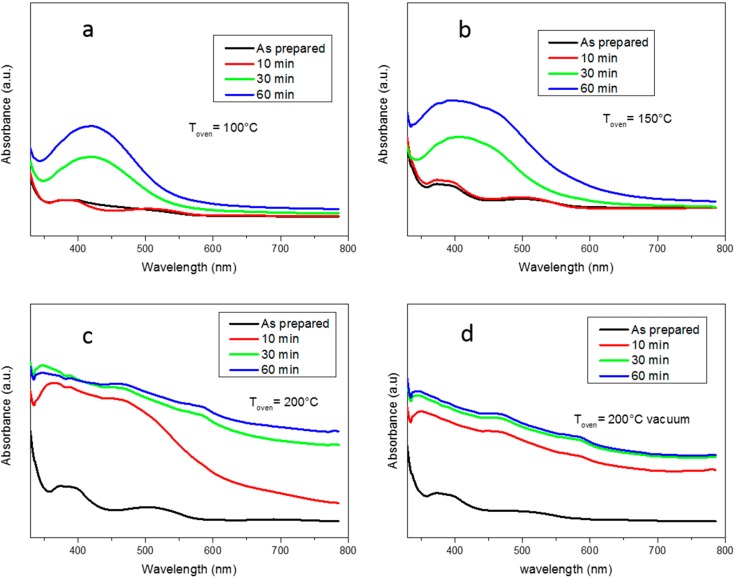
UV-Vis plots of samples treated at (**a**) 100 °C; (**b**) 150 °C; (**c**) 200 °C in air and (**d**) 200 °C in vacuum obtained after different heating times.

The kinetics of formation of silver nanoparticles has been followed by UV-Vis measurements (Perkin-Elmer Italia, Milano, Italy) performed on cured films exposed at different temperatures for increasing times. The formulations were irradiated for 10 s by visible light in order to mimic the curing process that occurs during DLP printing. Figure 4 reports the spectra obtained on four samples treated respectively at 100, 150, 200 °C in air and 200 °C in vacuum for different heating times, in order to follow the silver nanoparticles formation.

At first it is possible to observe that for all the samples almost no silver reduction occurred during visible irradiation. For the samples treated at 100 and 150 °C after 30 min of heating we can clearly see the appearance of the typical signal around 450 nm due to the plasmon of resonance of the metal NPs [47]. It is possible to evidence a progressive increase of the plasmon of resonance with the increase of heating time which means that the silver nanoparticles nucleation/growth is ongoing. While for the sample treated at 100 °C the appearing peak is relatively sharp, for the sample treated at 150 °C the peak is broader indicating the presence of bigger nanoparticles more closely packed; in fact, it is known [48] that the Surface Plasmon Resonance (SPR) phenomenon is related to the size and spacing of the nanoparticles.

When the sample treated at 200 °C in air is considered, it is visible that 10 min of heating are sufficient to obtain a broad SPR peak meaning that higher temperatures lead to a system in which silver NPs are numerous, with a broad dimensional distribution and closely packed. For longer heating times the absorption plot increases also at higher wavelengths; this corresponds to the appearance of the mirror-like aspect indicating the formation of a surface layer rich in NPs which strongly absorbs in the visible range. The same response is observable for the sample treated in vacuum even for shorter heating times (10 min).

**Figure 5:**
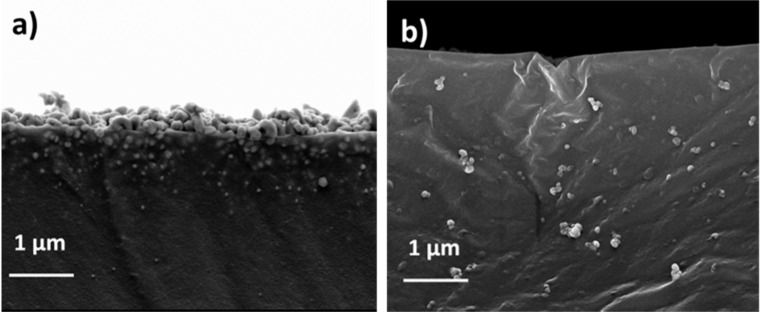

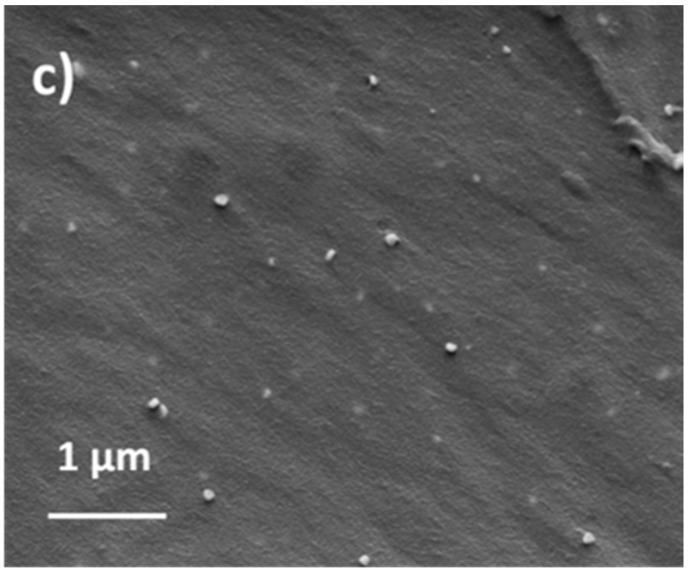
(**a**) Cross section of a sample treated 1 h at 150 °C in air; (**b**) Cross section of a sample treated 1 h at 150 °C in vacuum; (**c**) Cross section of the core of a sample treated 1 h at 150 °C in vacuum.

Shape and distribution of the silver nanoparticles into the printed polymeric matrices were investigated by Field Emission Scanning Electron Microscopy (FESEM) (Carl Zeiss AG, Jena, Germany). FESEM images of the 3D printed samples containing 15 phr AgNO_3_ after different thermal treatments are displayed in Figure 5; the formation of nanoparticles in the 3D structures is confirmed and, as can be observed from the figure, the silver nanoparticles are generally spherical in shape and relatively uniformly-dispersed inside the matrix. It is interesting to note that an important morphological difference clearly appears between the samples: Size, shape and distribution of silver nanoparticles into the matrix are different when the samples submitted to a post treatment at 200 °C in air (Figure 5a) or in vacuum (Figure 5b) are considered: In the first case, large unstructured aggregates are present at the surface, probably due to thermal oxidation (Figure 5a) while in the latter case smaller and more homogeneously dispersed nanoparticles are visible (Figure 5b).

Moreover, in both cases a relative enrichment of nanoparticles concentration could be observed at 3D printed structure surface (Figure 5b) with respect to the core of the structure (Figure 5c). This was already observed in some previous work [44,46] and could be explained with the general diffusion laws.

Differential scanning calorimetry (DSC) was also performed; the *T*_g_ values measured are reported in Table 1. It is possible to notice that a thermal treatment at higher temperatures for longer times induces a decrease of *T_g_*, related to polymer degradation observed in TGA experiments. In fact, the degradation leads to a general breakage of C–C bonds, reducing the chemical cross-linking points and thus to an increase of the mobility of the polymeric chains which corresponds to a decrease of glass transition temperature. On the contrary, a thermal treatment in harsh conditions but performed in nitrogen atmosphere does not induce a decrease of thermal properties since no degradation occurred, as demonstrated also in TGA experiments.

**Figure 6:**
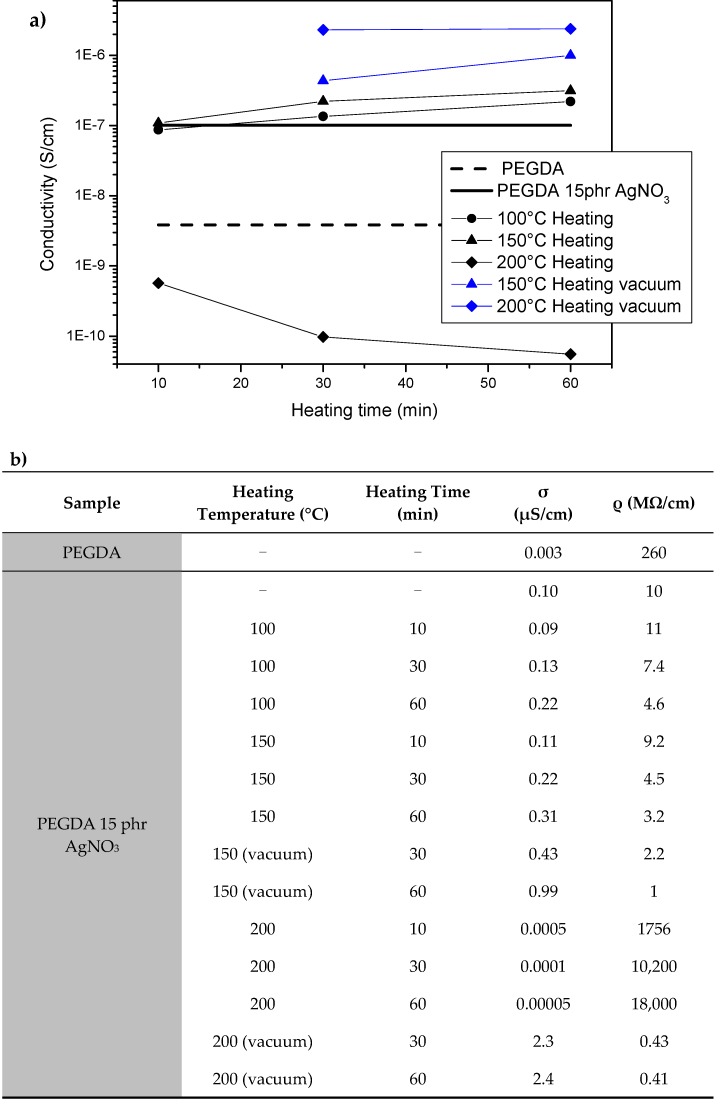
(**a**) Relationship between resistance and heating temperatures measured on flat printed specimens; (**b**) Conductivity and resistance values measured on flat samples treated at different temperatures for different times.

The electrical properties of the printed materials were then evaluated; firstly, the resistivity of flat printed specimens was measured by sandwiching them between copper electrodes. Figure 6a shows the relationship between the measured resistance and heating temperatures, the data are then reported in Figure 6b. The NC containing silver nitrate is two orders of magnitude more conductive than the neat polymeric matrix. This could be related to the presence of the conductive fillers dispersed in the polymeric matrix. Consequent thermal treatments in air condition up to a temperature of 150 °C, even for long periods of time, do not influence NCs resistivity, always keeping values of the order of some MΩcm. Those values are compatible with a hopping-controlled conduction mechanism [49], meaning that the NPs nucleation-growth mechanism induced by the temperature decreases the medium distance between the NPs, causing a moderate decrease of resistivity but not sufficient to guarantee an efficient percolating path.

It is important to observe that a thermal treatment at 200 °C performed in air involves a dramatically increase of the resistivity, this could be related to a large oxidation of silver NPs. On the contrary, similar thermal treatments performed in vacuum lead to the lower resistivity values, nearly two orders of magnitude lower than the corresponding untreated sample. The obtained values indicate that no sintering has occurred.

**Figure 7:**
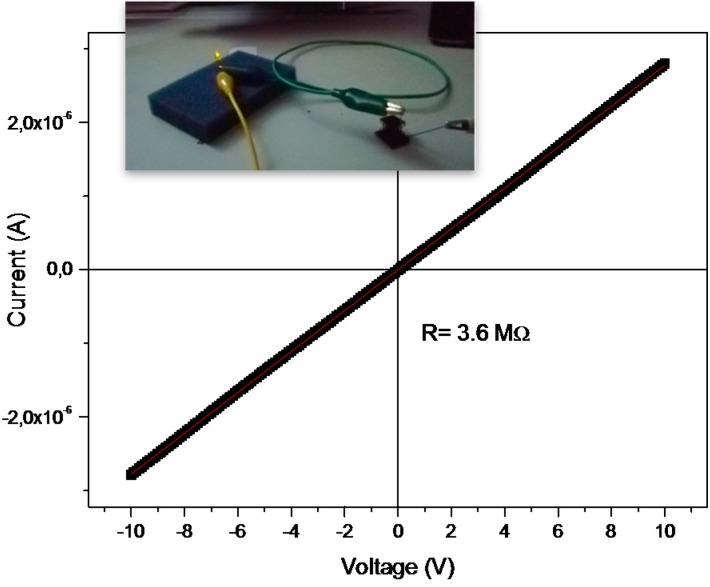
I–V plot obtained by contating an honeycomb structure treated at 200 °C in vacuum. Inset: the current flowing trough the structure was sufficient to achieve the illumination of a led.

In a recent work we reported about the fabrication of 3D printed object containing silver salts in which the subsequent Ag NPs reduction was performed by means of UV irradiation. The resistivity values obtained after 1 h of thermal treatment at 200 °C are in line with the values obtained for a sample UV irradiated for 10 min, indicating that with this NCs UV reduction seems more efficient in the realization of conductive 3D structures. At last we performed electrical measurements also on 3D structures, as example we reported the value for the sample heated at 200 °C for 1 h in vacuum, Figure 7. The current-voltage (I–V) curve results perfectly linear, showing a resistance of 3.6 MΩ. This value, although not very high, was sufficient to achieve the illumination of a led (Figure 6), thus envisaging a possible application in the market of electronics as dissipating material [50].

## 4. Conclusions

The method here proposed allows to obtain 3D structures with complex geometries presenting promising electrical properties. The in situ thermal generation of silver NPs performed on the printed samples does not influence the stability of the polymeric structures and represents a possible alternative to the UV generation already proposed.

## Figures and Tables

**Table 1:** *T*_g_ values measured in DSC for PEGDA containing 15 phr of AgNO_3_ after the thermal post treatment at different temperature (100 °C, 150 °C and 200 °C) at different time (10′, 30′ and 60′).

Treatment	*T*_g_ VALUE (°C)		
No TT	−28		
TT_Air	@100 °C	@150 °C	@200 °C
10′	−25	−25	−25
30′	−25	−26	−38
60′	−26	−30	−37
TT_Vacuum	-	@150 °C	@200 °C
30′	-	−26	−26
60′		−27	−28

## Abbreviations

The following abbreviations are used in this manuscript:

NPs	nanoparticles
TT	thermal treatment
3D	three-dimensional
NCs	nanocomposites
SLA	stereolithography
DLP	digital light processing
DMD	digital micromirror-array device
DSC	differential scanning calorimetry
TGA	thermogravimetric analysis
CAD	computer-aided design
BAPO	bis-acyl-phosphine oxide
PEGDA	polyethylene glycol diacrylate
FESEM	field emission scanning electron microscopy

## References

[1] Paul D.R., Robeson L.M. (2008). Polymer nanotechnology: Nanocomposites. Polymer.

[2] Hanemann T., Szabó D.V. (2010). Polymer-nanoparticle composites: From synthesis to modern applications. Materials.

[3] Tamborra M., Striccoli M., Comparelli R., Curri M.L., Petrella A., Agostiano A. (2004). Optical properties of hybrid composites based on highly luminescent cds nanocrystals in polymer. Nanotechnology.

[4] Lü C., Gao J., Fu Y., Du Y., Shi Y., Su Z. (2008). A ligand exchange route to highly luminescent surface-functionalized zns nanoparticles and their transparent polymer nanocomposites. Adv. Funct. Mater..

[5] Lu C., Yang B. (2009). High refractive index organic-inorganic nanocomposites: Design, synthesis and application. J. Mater. Chem..

[6] Koziej D., Fischer F., Kränzlin N., Caseri W.R., Niederberger M. (2009). Nonaqueous tio2 nanoparticle synthesis: A versatile basis for the fabrication of self-supporting, transparent, and uv-absorbing composite films. ACS Appl. Mater. Interfaces.

[7] Mutiso R.M., Kikkawa J.M., Winey K.I. (2013). Resistive switching in silver/polystyrene/silver nano-gap devices. Appl. Phys. Lett..

[8] Ling Q.-D., Liaw D.-J., Zhu C., Chan D.S.-H., Kang E.-T., Neoh K.-G. (2008). Polymer electronic memories: Materials, devices and mechanisms. Prog. Polym. Sci..

[9] Hedayati M., Faupel F., Elbahri M. (2014). Review of plasmonic nanocomposite metamaterial absorber. Materials.

[10] Gaharwar A.K., Peppas N.A., Khademhosseini A. (2014). Nanocomposite hydrogels for biomedical applications. Biotechnol. Bioeng..

[11] Schexnailder P., Schmidt G. (2009). Nanocomposite polymer hydrogels. Colloid Polym. Sci..

[12] Mallouki M., Tran-Van F., Sarrazin C., Simon P., Daffos B., De A., Chevrot C., Fauvarque J.F. (2007). Polypyrrole-Fe2O3 nanohybrid materials for electrochemical storage. J. Solid State Electrochem..

[13] Ingrosso C., Panniello A., Comparelli R., Curri M.L., Striccoli M. (2010). Colloidal inorganic nanocrystal based nanocomposites: Functional materials for micro and nanofabrication. Materials.

[14] Zhu W., Li J., Leong Y.J., Rozen I., Qu X., Dong R., Wu Z., Gao W., Chung P.H., Wang J. (2015). 3d-printed artificial microfish. Adv. Mater..

[15] Kim K., Zhu W., Qu X., Aaronson C., McCall W.R., Chen S., Sirbuly D.J. (2014). 3d optical printing of piezoelectric nanoparticle–polymer composite materials. ACS Nano.

[16] Sun K., Wei T.-S., Ahn B.Y., Seo J.Y., Dillon S.J., Lewis J.A. (2013). 3d printing of interdigitated li-ion microbattery architectures. Adv. Mater..

[17] Leigh S.J., Bradley R.J., Purssell C.P., Billson D.R., Hutchins D.A. (2012). A simple, low-cost conductive composite material for 3d printing of electronic sensors. PLoS ONE.

[18] Hofmann M. (2014). 3d printing gets a boost and opportunities with polymer materials. ACS Macro Lett..

[19] Quan Z., Wu A., Keefe M., Qin X., Yu J., Suhr J., Byun J.-H., Kim B.-S., Chou T.-W. (2015). Additive manufacturing of multi-directional preforms for composites: Opportunities and challenges. Mater. Today.

[20] Gou M., Qu X., Zhu W., Xiang M., Yang J., Zhang K., Wei Y., Chen S. (2014). Bio-inspired detoxification using 3d-printed hydrogel nanocomposites. Nat. Commun..

[21] Gross B.C., Erkal J.L., Lockwood S.Y., Chen C., Spence D.M. (2014). Evaluation of 3d printing and its potential impact on biotechnology and the chemical sciences. Anal. Chem..

[22] Petrovic V., Vicente Haro Gonzalez J., Jordá Ferrando O., Delgado Gordillo J., Ramón Blasco Puchades J., Portolés Griñan L. (2011). Additive layered manufacturing: Sectors of industrial application shown through case studies. Int. J. Prod. Res..

[23] Wang X., Yan Y., Zhang R. (2007). Rapid prototyping as a tool for manufacturing bioartificial livers. Trends Biotechnol..

[24] Chan V., Jeong J.H., Bajaj P., Collens M., Saif T., Kong H., Bashir R. (2012). Multi-material bio-fabrication of hydrogel cantilevers and actuators with stereolithography. Lab Chip.

[25] Ladd C., So J.-H., Muth J., Dickey M.D. (2013). 3d printing of free standing liquid metal microstructures. Adv. Mater..

[26] Chiappone A., Fantino E., Roppolo I., Lorusso M., Manfredi D., Fino P., Pirri C.F., Calignano F. (2016). 3d printed peg-based hybrid nanocomposites obtained by sol–gel technique. ACS Appl. Mater. Interfaces.

[27] Schultz A.R., Lambert P.M., Chartrain N.A., Ruohoniemi D.M., Zhang Z., Jangu C., Zhang M., Williams C.B., Long T.E. (2014). 3d printing phosphonium ionic liquid networks with mask projection microstereolithography. ACS Macro Lett..

[28] Peterson G.I., Larsen M.B., Ganter M.A., Storti D.W., Boydston A.J. (2015). 3d-printed mechanochromic materials. ACS Appl. Mater. Interfaces.

[29] Wang X., Guo Q., Cai X., Zhou S., Kobe B., Yang J. (2014). Initiator-integrated 3d printing enables the formation of complex metallic architectures. ACS Appl. Mater. Interfaces.

[30] Vatani M., Lu Y., Engeberg E.D., Choi J.-W. (2015). Combined 3d printing technologies and material for fabrication of tactile sensors. Int. J. Precis. Eng. Manuf..

[31] Nassar I.T., Weller T.M. An electrically-small, 3-d cube antenna fabricated with additive manufacturing. Proceedings of the 2013 IEEE Topical Conference on Power Amplifiers for Wireless and Radio Applications (PAWR).

[32] Lee M.P., Cooper G.J.T., Hinkley T., Gibson G.M., Padgett M.J., Cronin L. (2015). Development of a 3d printer using scanning projection stereolithography. Sci. Rep..

[33] Corcione C.E., Greco A., Maffezzoli A. (2006). Temperature evolution during stereolithography building with a commercial epoxy resin. Polym. Eng. Sci..

[34] Esposito Corcione C., Greco A., Maffezzoli A. (2004). Photopolymerization kinetics of an epoxy-based resin for stereolithography. J. Appl. Polym. Sci..

[35] Corcione C.E. (2014). Development and characterization of novel photopolymerizable formulations for stereolithography. J. Polym. Eng..

[36] Gibson I., Rosen D., Stucker B. (2015). Vat photopolymerization processes. Additive Manufacturing Technologies: 3d Printing, Rapid Prototyping, and Direct Digital Manufacturing.

[37] Czyżewski J., Burzyński P., Gaweł K., Meisner J. (2009). Rapid prototyping of electrically conductive components using 3d printing technology. J. Mater. Process. Technol..

[38] Leigh S.J., Purssell C.P., Bowen J., Hutchins D.A., Covington J.A., Billson D.R. (2011). A miniature flow sensor fabricated by micro-stereolithography employing a magnetite/acrylic nanocomposite resin. Sens. Actuators A Phys..

[39] Yugang D., Yuan Z., Yiping T., Dichen L. (2011). Nano-tio 2 -modified photosensitive resin for rp. Rap. Prototyp. J..

[40] Kalsoom U., Peristyy A., Nesterenko P.N., Paull B. (2016). A 3d printable diamond polymer composite: A novel material for fabrication of low cost thermally conducting devices. RSC Adv..

[41] Licciulli A., Corcione C.E., Greco A., Amicarelli V., Maffezzoli A. (2004). Laser stereolithography of ZrO_2_ toughened Al_2_O_3_. J. Eur. Ceram. Soc..

[42] Scalera F., Esposito Corcione C., Montagna F., Sannino A., Maffezzoli A. (2014). Development and characterization of uv curable epoxy/hydroxyapatite suspensions for stereolithography applied to bone tissue engineering. Ceram. Int..

[43] Gmeiner R., Mitteramskogler G., Stampfl J., Boccaccini A.R. (2015). Stereolithographic ceramic manufacturing of high strength bioactive glass. Int. J. Appl. Ceram. Technol..

[44] Fantino E., Chiappone A., Roppolo I., Manfredi D., Bongiovanni R., Pirri C.F., Calignano F. (2016). 3d printing of conductive complex structures with in situ generation of silver nanoparticles. Adv. Mater..

[45] Rajan K., Roppolo I., Chiappone A., Bocchini S., Perrone D., Chiolerio A. (2016). Silver nanoparticle ink technology: State of the art. Nanotechnol. Sci. Appl..

[46] Roppolo I., Doriguzzi Bozzo A., Castellino M., Chiappone A., Perrone D., Bejtka K., Bocchini S., Sangermano M., Chiolerio A. (2016). Dual step irradiation process for in situ generation and patterning of silver nanoparticles in a photocured film. RSC Adv..

[47] Stamplecoskie K.G., Scaiano J.C. (2012). Silver as an example of the applications of photochemistry to the synthesis and uses of nanomaterials. Photochem. Photobiol..

[48] Henry A.-I., Bingham J.M., Ringe E., Marks L.D., Schatz G.C., Van Duyne R.P. (2011). Correlated structure and optical property studies of plasmonic nanoparticles. J. Phys. Chem. C.

[49] Krishnan K., Tsuruoka T., Mannequin C., Aono M. (2016). Mechanism for conducting filament growth in self-assembled polymer thin films for redox-based atomic switches. Adv. Mater..

[50] Chiolerio A., Roppolo I., Sangermano M. (2013). Radical diffusion engineering: Tailored nanocomposite materials for piezoresistive inkjet printed strain measurement. RSC Adv..

